# Separation of Hafnium From Zirconium by Anion Exchange

**DOI:** 10.6028/jres.065A.008

**Published:** 1961-02-01

**Authors:** John L. Hague, Lawrence A. Machlan

## Abstract

The results of a systematic survey of the elution of hafnium and zirconium in diluted sulfuric acid, and in solutions of hydrochloric and sulfuric acids in water, are presented. The data characterize the behavior of the sulfate anion complexes of these elements on a strong quaternary-amine anion-exchange resin column. The possibility of separating these elements is demonstrated.

## 1. Introduction

G. Hevesy has written that [6]^1^ “In view of the great similarity between hafnium and zirconium, it is hardly possible to separate these two elements in a single operation.” A simple one-step separation adequate for analytical work has still to be developed for hafnium-zirconium mixtures, although recent work employing ion exchange has developed suitable methods for the separation of niobium from tantalum [5, 9, 10] and of molybdenum from tungsten [2, 9, 10], and a promising start has even been made on separating the rare earths [12].

Previous work on zirconium-hafnium mixtures has shown that fractionation on anion-exchange resins can be obtained by elution with aqueous solutions of hydrochloric and hydrofluoric acids [7, 8]; or by using diluted sulfuric acid, or nitric-citric acid solutions on cation-exchange resin-columns [1, 11]. Preliminary work has shown that it is not feasible, within convenient column parameters and eluant acidities, to obtain a one-step separation in a hydrochloric acid medium, in hydrochloric-hydrofluoric acid solutions, or in sulfuric-hydrofluoric acid solutions.

Several different hydrochloric-sulfuric acid solutions appeared to provide usable separations, but difficulties were encountered which appeared to be associated with the polymerization and eventual hydrolysis of zirconium ions. The results of experiments with sulfuric acid solutions indicate that a satisfactory separation of hafnium from zirconium can be obtained.

## 2. Experimental Procedures

### 2.1. Ion-Exchange Columns

The columns used in these experiments were constructed of polystyrene tubes approximately 12 in. long and 1 in. i.d. A simple column can be prepared from such tubing as follows: Insert a piece of polystyrene tubing 6 in. long, 316 in. o.d. (with a 116 in. wall) through a No. 5 waxed, rubber stopper, so that one end of the tubing is flush with the small end of the stopper. Attach another 6 in. length of the same tubing with a 2-in. length of Tygon R tubing. Close the bottom of the one-in. i.d. tube by inserting the rubber stopper. Attach a hose-cock clamp, used to control the flow rate, to the Tygon tubing. Columns suitable for continuous operation have previously been described for both glass [3] and plastic [4] assemblies, and these are convenient to use if a number of analyses are to be performed.

The anion-excliange resins used were Dowex–1, 200- to 400-mesh, having 8- or 10-percent of divinylbenzene crosslinkages. Experience during several years has shown that the mesh size of these resins may vary considerably from lot to lot. To select resin of suitable size, the material as received is air-dried, and sieved through a 270-mesh sieve. Most of the very fine material is removed from the fraction passing the 270-mesh sieve, as follows: Prepare a suspension of the resin in diluted^2^ hydrochloric acid (1 + 19). The coarser fraction is allowed to settle for 10 to 15 minutes, and the fines removed by decantation. Repeat the process several times, until most of the very fine material is removed from the suspension.

Load the column with resin as follows: Cover the bottom of the column with a ¼- to ⅜-in. layer of acid-resistant, polyvinyl chloride plastic “wool.” Add portions of the resin suspension so as to obtain a settled column of the resin that is 6- to 7-in. high. Wash the loaded column with approximately 100 ml of diluted nitric acid (1 + 9), and then perform several elution cycles by alternate additions of diluted hydrochloric acid (1 + 9) and diluted hydrochloric acid (3 + 1) to remove the remaining fines. Finally, wash the column with diluted sulfuric acid (1 + 19), to convert the resin to the sulfate form. Approximately 350 to 400 ml of sulfuric acid solution will be required, and the removal of the chloride can be followed by testing portions of the eluate with silver nitrate solution. The resin charge should not be allowed to become dry.

### 2.2. Elution Procedure

Dilute acid solutions which covered the range of 1 to 3 percent by volume of hydrochloric acid with sulfuric acid in the range of 1 to 3.5 percent by volume were used. Dilute sulfuric acid solutions were also used in the range of 2 to 10 percent by volume. For each combination of acids, or each concentration of sulfuric acid, elution was continued until column equilibration was obtained. Usually, 300 to 500 ml of eluant was required, and the progress of the equilibration was followed with sufficient accuracy by titration of the acid in an aliquot of the eluate.

The zirconium and hafnium solutions were prepared by dissolving the “reactor grade” metals, usually about 50 mg, in sulfuric-hydrofluoric acid solutions. The zirconium was nearly hafnium-free, but the hafnium contained several percent of zirconium. The solution was evaporated several times to fumes of sulfuric acid, and the walls of the crucible were washed down with water between fumings. The amount of sulfuric acid used was so chosen as to give the same concentration in 50 ml as was present in the particular eluant being tested. For those solutions which were to contain mixed acids, the proper amount of hydrochloric acid was added before final dilution.

After dilution to 50 ml, the solution was added to the equilibrated ion-exchange column. The first 25 ml of eluate was discarded, and 50-ml fractions of eluate were collected during the addition of eluant. These fractions were so adjusted as to contain approximately 15 percent of hydrochloric acid, by volume, in 200 ml, and then cooled to 5 °C. The zirconium or hafnium was precipitated with cupferron. The ignited oxide obtained from each fraction was weighed and calculated to a percentage of the total amount, and the accumulated percentages were plotted against volume of eluate.

## 3. Results

### 3.1. Elution With Diluted Hydrochloric-Sulfuric Acid Solutions

**Figure 1:**
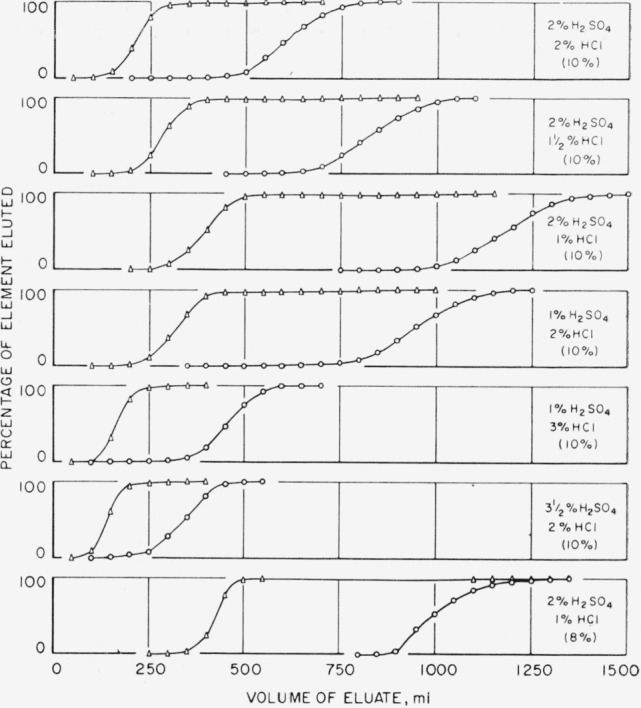
Elution behavior of hafnium and zirconium in solutions of hydrochloric and sulfuric acids. (Δ “Hafnium” solution; o zirconium.)

**Figure 2:**
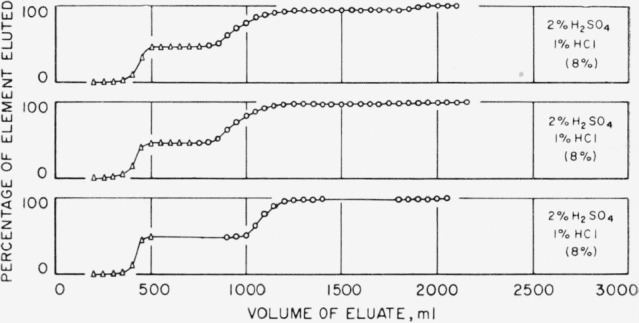
Separation of hafnium-zirconium mixtures in hydrochloric-sulfuric acid solutions.

The results of a series of experiments covering the range of hydrochloric-sulfuric acid concentrations of interest in the separation of hafnium-zirconium mixtures are shown in figure 1. The solution containing 2 percent by volume of sulfuric acid and 1 percent by volume of hydrochloric acid seemed to offer some promise of a separation, but the “sum of the oxides” recovered was always a little low. Figure 2 shows the results obtained and that, at approximately 2 liters, a third fraction appeared which contained the missing fraction of the elements. The first and second curves show the behavior of mixtures of approximately 100 mg of each element, and the third curve is for approximately 25 mg of each element. The extra fraction was examined spectrographically and found to be very strong in both hafnium and zirconium, but the composition has not been unequivocally established, because of the possibility of cross contamination due to the sequence of runs on the column. The procedure, as a simple separation suitable for analytical work, was abandoned. It might prove of interest to those concerned with the polymerization and hydrolysis of zirconium and hafnium ions in solutions of this nature.

### 3.2. Elution With Sulfuric Acid

**Figure 3:**
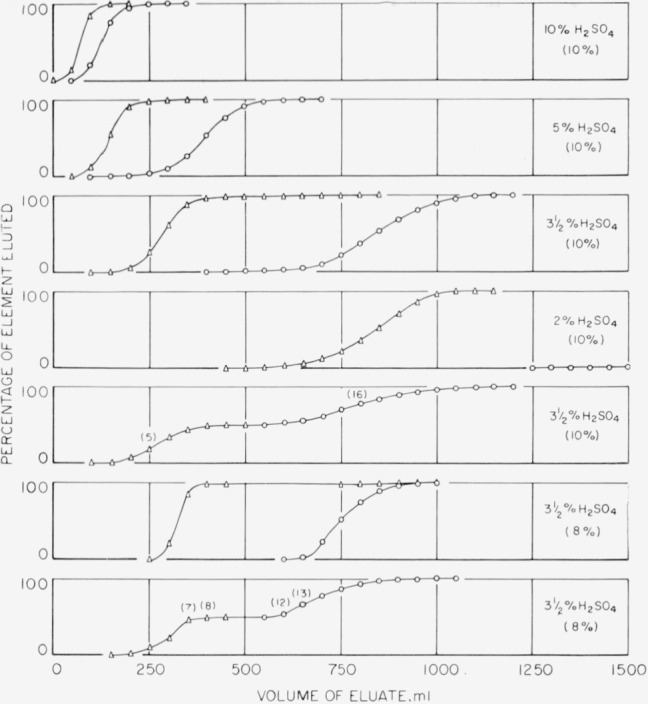
Elution behavior and separation of hafnium from zirconium. (Δ “Hafnium” solution; o zirconium.)

The results of our study of the behavior of zirconium and hafnium when eluted with solutions containing sulfuric acid are given in figure 3, except for zirconium in 2-percent sulfuric acid, which was nearly, but not completely, eluted at 3 liters. The “tail” on the hafnium curves presumably represents the zirconium content of the hafnium used in the experiments. Resin having 10 percent of divinvlbenzene crosslinkages and consisting of particles of up to 200-mesh size (about 75 *μ*), was used in obtaining the first five curves in figure 3. The column and preparation of the resin have been described previously [2]. The fifth curve shows the results of an attempt to separate a mixture prepared from 100 mg each of hafnium and zirconium. Spectrographic examination of fraction 5 indicated hafnium with an undetectable amount of zirconium, and, of fraction 16, showed zirconium with an undetectable amount of hafnium. Thus, a good fractionation of hafnium from zirconium resulted, but a clear-cut line of demarcation between the hafnium and zirconium fractions was not observed.

Experience [4, 9] has shown that a separation can frequently be improved by proper adjustment of column parameters. Accordingly, a column was prepared by use of resin with 8 percent of divinylbenzene crosslinkages having a maximum particlesizing of about 50 *μ* (passing through a 270-mesh sieve). A settled bed of resin, approximately 7.5 in. high, was prepared, and elution was at a flow rate of about 100 ml per hour, instead of the 125 ml per hour previously used. Further experiments on the elution of hafnium and zirconium in 3.5-percent sulfuric acid (line 6 of fig. 3) indicated a considerable “sharpening” of the separation.

The last elution curve in figure 3 illustrates the separation of hafnium from zirconium (approximately 100 mg of each). Approximately 150 ml of eluate containing neither element was obtained between the respective eluates of the two. A spectrographic examination of fractions 7 and 8 indicates that these hafnium fractions contained 10 to 100 ppm of zirconium. Similar examination of fraction 12 showed a composition of zirconium containing 10 to 100 ppm of hafnium; in fraction 13 hafnium was not identified with certainty. Since these small fractions close to each other are those most likely to exhibit cross-contamination, the separation is excellent and well suited to analytical applications.

A study of the elution of other elements under these conditions has not yet been made, although a limited number of papers on the elution of sulfates now exists [10]. The precipitation procedure employing mandelic acid or phosphate provide a reasonably specific step for concentration of zirconium and hafnium. Work is in progress to apply the separation to mixtures of the elements such as exist in “nuclear grade” hafnium, and to other mixtures of interest.

## Footnotes

1 Figures in brackets indicate the literature references at the end of this paper.

2 Diluted hydrochloric acid (1+19) denotes 1 volume of concentrated hydrochloric acid, sp gr 1.18, diluted with 19 volumes of water. If no dilution is specified, the concentrated analytical reagent is meant.
